# Virome diversity of *Hyalomma dromedarii* ticks collected from camels in the United Arab Emirates

**DOI:** 10.14202/vetworld.2023.439-448

**Published:** 2023-03-16

**Authors:** Nighat Perveen, Biduth Kundu, Naganeeswaran Sudalaimuthuasari, Raja Saeed Al-Maskari, Sabir Bin Muzaffar, Mohammad Ali Al-Deeb

**Affiliations:** 1 Department of Biology, United Arab Emirates University, Al-Ain, P.O. Box 15551, UAE; 2 Khalifa Center for Genetic Engineering and Biotechnology, United Arab Emirates University, Al Ain, P.O. Box 15551, UAE

**Keywords:** camels, *Hyalomma dromedarii*, nanopore technology, UAE, viral diversity, virome analysis, whole genome sequencing

## Abstract

**Background and Aim:**

Viruses are important components of the microbiome of ticks. Ticks are capable of transmitting several serious viral diseases to humans and animals. Hitherto, the composition of viral communities in *Hyalomma dromedarii* ticks associated with camels in the United Arab Emirates (UAE) remains unexplored. This study aimed to characterize the RNA virome diversity in male and female *H. dromedarii* ticks collected from camels in Al Ain, UAE.

**Materials and Methods:**

We collected ticks, extracted, and sequenced RNA, using Illumina (NovaSeq 6000) and Oxford Nanopore (MinION).

**Results:**

From the total generated sequencing reads, 180,559 (~0.35%) and 197,801 (~0.34%) reads were identified as virus-related reads in male and female tick samples, respectively. Taxonomic assignment of the viral sequencing reads was accomplished based on bioinformatic analyses. Further, viral reads were classified into 39 viral families. Poxiviridae, Phycodnaviridae, Phenuiviridae, Mimiviridae, and Polydnaviridae were the most abundant families in the tick viromes. Notably, we assembled the genomes of three RNA viruses, which were placed by phylogenetic analyses in clades that included the Bole tick virus.

**Conclusion:**

Overall, this study attempts to elucidate the RNA virome of ticks associated with camels in the UAE and the results obtained from this study improve the knowledge of the diversity of viruses in *H. dromedarii* ticks.

## Introduction

Ticks are hematophagous ectoparasites that can transmit a wide range of microbial pathogens and substantially threaten animal and human health [1, 2]. Genomic analyses through next-generation sequencing platforms have proven valuable for investigating the diversity and composition of microbes in ticks [3]. The discovery of novel pathogens may be facilitated by the characterization of the tick microbiome, which is important to understand the relationship between microbes and their tick hosts [4]. Tick viromes have not been well-studied and high-throughput sequencing facilitates the investigation of diversity and circulation of existing and novel viruses in tick vectors [5]. In recent decades, several viral epidemics have been reported in the Arabian Peninsula [6–8], although the knowledge about the viruses in the region remains limited. Comprehensive surveillance and screening of vector populations, especially ticks, are essential to prepare for new viral outbreaks. Recently, the Middle East and North Africa (MENA) region has observed the emergence and re-emergence of several viruses [6, 9–15]. Notable examples include the Middle East Respiratory Syndrome virus [9, 10], Alkhurma hemorrhagic fever virus [11], Crimean-Congo hemorrhagic fever virus (CCHFV) [12, 13], and Kadam virus [6]. Recent studies screening for anti-CCHFV antibodies indicated that CCHFV likely persisted in the UAE, suggesting widespread surveillance of this tick-borne virus is needed [14, 15]. Several studies have reported novel viruses outside the MENA region in ticks. For example, new viruses were discovered from *Ixodes scapularis*, *Dermacentor variabilis*, and *Amblyomma americanum* collected in New York and Pennsylvania [16–18], which include monoegavirus, nairoviruses, and phleboviruses [16]. The pathogenicity and the impact of most of these viruses remain unstudied.

The UAE has a camel population of approximately 459,000 camel heads [19]. *Hyalomma dromedarii* ticks feed mainly on blood from camels and can transmit viruses such as the CCHFV, Dera–Ghazi–Khan virus, Dhori virus, and Kadam virus [6, 20, 21]. Recently, a new lineage of CCHFV was isolated from dromedary camels in UAE. S segment analysis of the CCHFV genome showed the highest percentage nucleotide (NT) identity at 89.8% with a CCHFV isolate (from humans) from South Africa (KJ682821) and was an outlier to the Africa 3 lineage. Furthermore, M segment analysis placed the UAE CCHFV into a distinct lineage, Asia 4, due to possible reassortment of the current sequence from camel and its nearest neighbor (76.7% NT identity) from a tick in Pakistan (KY484038). Furthermore, L segment analysis showed the highest percentage identity (NT identity at 87.7%) with a human CCHFV isolate from Russia (KX013486) [15]. Camels are frequently brought from different parts of Asia and Africa into the Middle East. This could result in widespread mixing of viral lineages leading to the emergence of novel variants [15]. The patterns of tick–host–virus interrelationships are changing due to changes in population densities of ticks, hosts, and other environmental factors [22]. An understanding of the epidemiology of tick-borne viral diseases, tick vector competence, and transmission dynamics in tick vectors is important for designing tick management strategies and curtailing the spread of viral infections.

The composition of viral communities in *H. dromedarii* ticks in the UAE is not adequately explored and therefore, there is a need for studies to quantify tick-borne viral diversity and abundance. This study aimed to characterize the virome diversity of male and female *H. dromedarii* ticks collected from Al Ain, UAE.

## Materials and Methods

### Ethical approval

Tick collection was carried out in accordance with the recommendations of the Animal Research Ethics Committee of the UAE University (ethical approval# ERA_2019_5953). In addition, the experimental protocol was approved by the UAE University Research Office.

### Study period and location

Ticks were collected during the month of June 2021 from camel farms in Al Ain (24.1302° N, 55.8023° E), UAE. Ticks were processed in Animal Ecology and Entomology Laboratory at United Arab Emirates University, UAE.

### Tick sampling and identification

Live ticks were collected from 15 camels in Al Ain, UAE and were frozen immediately in liquid nitrogen. From each camel, ticks were collected manually from the whole body and 10 ticks were collected per animal. Therefore, a total of 150 ticks were collected. All the collected ticks were taken to the Animal Ecology and Entomology Laboratory at UAE University and identified morphologically using tick taxonomic keys [23, 24]. For molecular identification, our previously published protocol was followed [25]. Briefly, legs from ticks were removed with a sterile scalpel blade and homogenized using liquid nitrogen in a 1.5 mL tube. The DNA extraction was accomplished by using DNA Mini Kit (Qiagen, Hilden, Germany) following the manufacturer’s protocol. The isolated DNA quality was confirmed using 1% agarose gel electrophoresis and DNA concentration was measured using a NanoDrop 2000 UV spectrophotometer (Thermo Fisher Scientific, Waltham, MA, USA). We used the 16S rRNA gene-specific oligonucleotide primer pair (16S + 1: CTGCTCAATGATTTTTTAAATTGCTGTGG, 16S − 1: CCGGTCTGAACTCAGATCAAGT) to amplify 460 bp based on a published protocol [26]. After polymerase chain reaction (PCR), sequencing of the amplicon was performed on Genetic Analyzer 3500 (using primers 16S + 1 and 16S − 1). Further, a sequenced amplicon similarity search was carried out using the online BLAST tool (https://blast.ncbi.nlm.nih.gov) in GenBank NCBI.

### Whole RNA isolation, quality control, pooling, and sequencing

A total of 150 ticks were collected from 15 camels at a farm in Al Ain in the eastern region of Abu Dhabi, UAE (24°01’38.7”N 55°38’57.8”E). After the identification of ticks, male and female ticks were washed using Dulbecco’s phosphate-buffered saline solution by following the protocol detailed in Harvey *et al*. [27]. For each RNA extraction, two male and female ticks were used from each camel sample (10 ticks were collected from each camel). Accordingly, 30 male and 30 female ticks (out of 150 ticks from 15 animals) were subjected to RNA extraction. After RNA extraction from each tick, we created two pools, one for male ticks and one for female ticks (all RNAs for male ticks were pooled together in one tube and all RNAs from female ticks were pooled together in another tube). Ticks were homogenized and high-quality whole RNA was isolated using the Promega Maxwell RSC simplyRNA Tissue kit (Promega, Madison, WI, USA), based on the manufacturer’s instructions. Isolated RNA quality and quantity were confirmed using agarose gel electrophoresis, NanoDrop 2000 spectrophotometer (ThermoFisher Scientific™, Waltham, MA, USA), and Qubit 2.0 fluorometer (ThermoFisher Scientific™). Samples were pooled for sequencing into male and female pools. Furthermore, RNAs (from the same subset of ticks) was used for both Illumina and Oxford Nanopore Technology based library method. Before library preparation, the host rRNAs were removed using RiboMinus Eukaryote Kit (Cat no. A10837-08, Thermo Fisher Scientific), then the RNAs were converted into cDNA and Illumina compatible RNAseq library was prepared using NEBNext Ultra Directional RNA Library Prep Kit (New England Biolabs, Ipswich, MA, USA) and sequenced using Illumina Novaseq 6000 machine (150 bp PE chemistry). We prepared the Oxford Nanopore – MinION compatible libraries using cDNA-PCR sequencing Kit (Cat no. SQK-PCS 109, Oxford Nanopore, Abingdon, UK) and sequenced them in MinION sequencer (flow cell id: FLO-MIN106D R9.4 revision D chip).

### Sequencing data quality control and trimming

The initial raw Illumina data quality was confirmed using FastQC tool (https://www.bioinformatics.babraham.ac.uk/projects/fastqc/) and, adapter and low-quality regions present in the reads were trimmed using Trimmomatic v.0.39 software [28]. Raw data generated from the MinION were error corrected and trimmed using CANU v.2.1.1 [29].

### Virus profiling based on illumina reads

Illumina reads from male and female tick samples were searched against the virus reference genome database using Kaiju v.1.8.0 (−z 50 −e 1 −m 20 −E 0.001) [30] and possible virus-related reads were taxonomically classified. Based on the virus taxonomy classification, virus family distribution Krono diagram was created with KronoTools v.2.7.1 [31]. Similarly, reads were searched against nr database and fungi database and used to identify the distribution of eukaryotic, fungi, and bacteria-related reads.

### Virus genome assembly and annotation

Trimmed Illumina and Nanopore reads were used for hybrid *de novo* assembly using rnaSpades v.3.15.3 program (parameter: −t 80 −m 700) [32]. Assembled contigs were further error corrected using Pilon v.1.23 tool [33]. Corrected contigs were searched against the virus reference genome database using the kaiju program and contigs showing similarity with virus genomes were extracted for the downstream annotation.

### Virus genome annotation

The complete genome found in our assembly was annotated using the GeneMarkS program [34]. The phylogenetic tree of the related virus was created using the neighbor-joining method using MEGA–X software (https://www.megasoftware.net/) with the bootstrap value of 1000 replicates.

### Accession number(s)

A representative sequence of *H. dromedarii* was submitted to GenBank and acquired accession number ON937757. All sequencing data generated during this study were submitted to the NCBI-SRA database under the BioProject ids PRJNA827495 (female), and PRJNA844213 (male) (Table-1). All assembled virus sequences were deposited in the Zenodo data repository (https://zenodo.org/).

**Table-1:** Information on the library constructed and data generated in this study.

Library ID	Sample site	Host	Collection date	No. of ticks	No. of total reads	No. of bases	RNAseq technique	NCBI SRA accession no.
HDM1*	Al Ain	Camel ♀	June-2021	30 ♂	51,994,138	15.6 Gb	Illumina NovaSeq 6000 (150bp×2)	SRR19500736
HDM2**	Al Ain	Camel ♀	June-2021	30 ♂	1,000,182	553.3 Mb	Oxford Nanopore (MinION)	SRR19500735
HDF1***	Al Ain	Camel ♀	June-2021	30 ♀	58,119,014	17.4 Gb	Illumina NovaSeq 6000 (150bp×2)	SRR18797880
HDF2****	Al Ain	Camel ♀	June-2021	30 ♀	707,067	267.3 Mb	Oxford Nanopore (MinION)	SRR18797879

* HDM1: *Hyalomma dromedarii* Male 1 (for Illumina NovaSeq 6000),

** HDM2: *Hyalomma dromedarii* Male 2 (for Oxford Nanopore [MinION]),

*** HDF1: *Hyalomma dromedarii* Female 1 (for Illumina NovaSeq 6000),

**** HDF2: *Hyalomma dromedarii* Female 2 (for Oxford Nanopore [MinION]). ♀: Female, ♂: Male

## Results

### Tick species identification

We analyzed 150 ticks collected at Al Ain, Abu Dhabi, UAE. All the ticks were identified as *H*. *dromedarii* morphologically. This identification was confirmed using the 16S rRNA gene which showed 99.75% similarity between the UAE sample and *H. dromedarii* GenBank samples from Pakistan (OL307008, coverage: 95% and E-valve: 0.0), India with 99.50% similarity (LC661680, coverage: 95% and E-valve 0.0), and UAE with 99.50% similarity (MZ976772, coverage: 95% and E-valve 0.0).

### Virome analysis

Based on the morphological identification, the collected ticks were separated into two types of samples; male (30 ticks) and female (30 ticks). In total 51,994,138 (~15.6 Gb data) and 1,000,182 (553.3 Mb data) reads were generated from the male sample using Illumina and MinION, respectively. Similarly, 58,119,014 (17.4 Gb data) and 707,067 (267.3 Mb data) sequencing reads, respectively, were generated from the female sample (Table-1).

Taxonomic classification of the sequencing data resulted in 180,559 (~0.35%) and 197,801 (~0.34%) virus-related reads in male and female tick samples, respectively. Overall, ~5% bacterial, ~1.5% fungal, and ~93% of animal (ticks and camel) related reads were identified in the male sample. In the female sample, ~12% bacterial, ~1% fungal, and ~86% of animal (ticks and camel) related reads were profiled.

**Figure-1:**
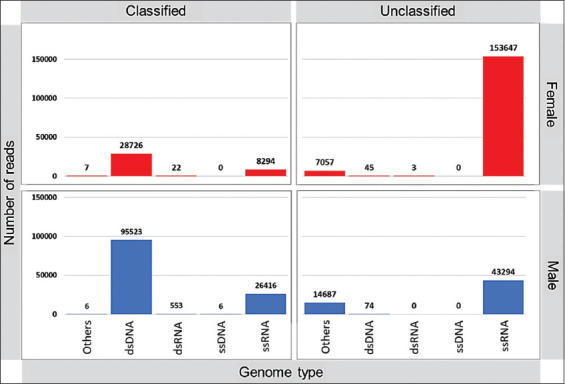
Overview of the number of viral reads grouped by viral type.

Analysis of the viral reads obtained from female tick samples revealed that 77.7% were similar to unclassified ssRNA viruses, 14.5% to classified dsDNA viruses, 4.12% were similar to classified ssRNA viruses, and <1% unclassified dsDNA viruses, dsRNA viruses or unclassified dsRNA viruses (Figure-1). However, the viral reads obtained from male ticks revealed that 52.9% were similar to classified dsDNA viruses, 24% to unclassified ssRNA viruses, 14.6% to classified ssRNA viruses, and <1% unclassified dsDNA viruses and classified dsRNA viruses (Figure-1).

Taxonomic profiling of viruses revealed that at the kingdom, phylum, and class levels, the maximum viral reads belonged to *Bamfordvirae*, *Nucleocytoviricota*, and *Megaviricetes*, in both male and female ticks. However, *Chitovirales* was the most abundant order in male ticks and *Algavirales* in female ticks.

**Figure-2:**
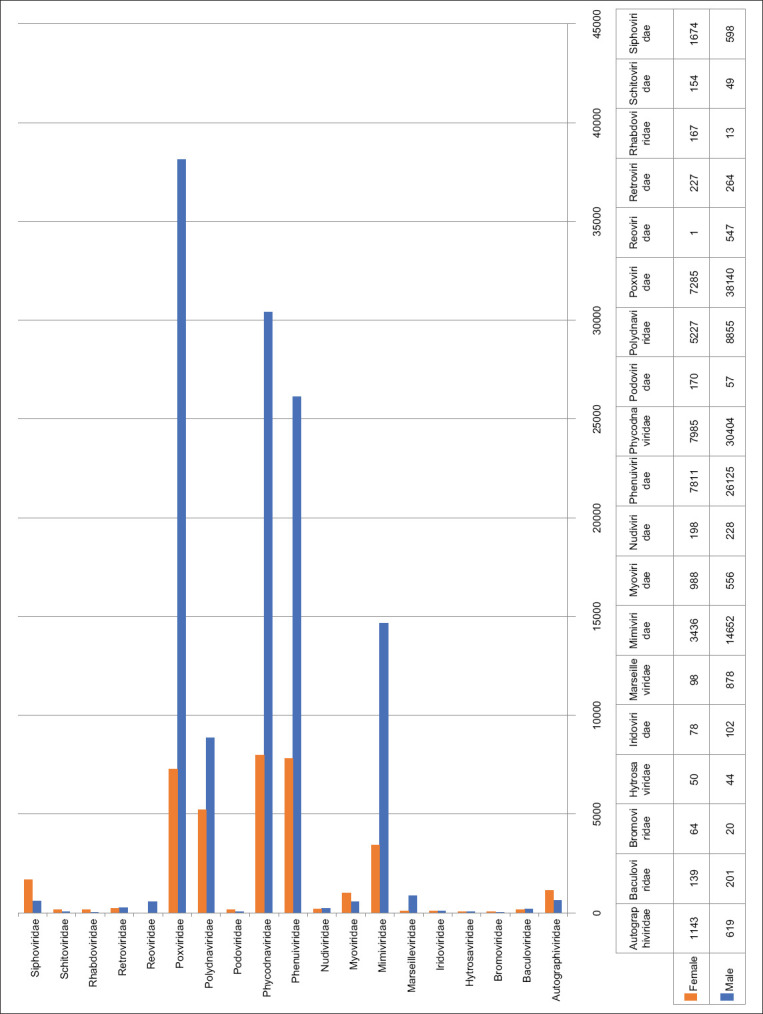
The number of viral reads at the family level obtained from classified viruses.

Viral sequences were classified into 39 viral families. Other viral sequences shared by all unclassified *Riboviria*, unclassified *Varidnaviria*, and unclassified *Duplodnaviria*. *Poxviridae* (21%), *Phycodnaviridae* (16.8%), *Phenuiviridae* (14.5%), *Mimiviridae* (8.1%), and *Polydnaviridae* (4.9%) were the most abundant families in the male tick viromes (Figure-2). Similarly, *Phycodnaviridae* (4%)*, Phenuiviridae* (3.9%), and *Poxviridae* (3.7%) were the most abundant families in the female tick viromes (Figure-2). *Siphoviridae*, *Autographiviridae*, and *Myoviridae* were found abundant in female ticks as compared to male ticks, whereas *Marseilleviridae* and *Reoviridae* were abundant in male ticks (Figure-2). Most of these abundant families are dsDNA virus families.

**Figure-3:**
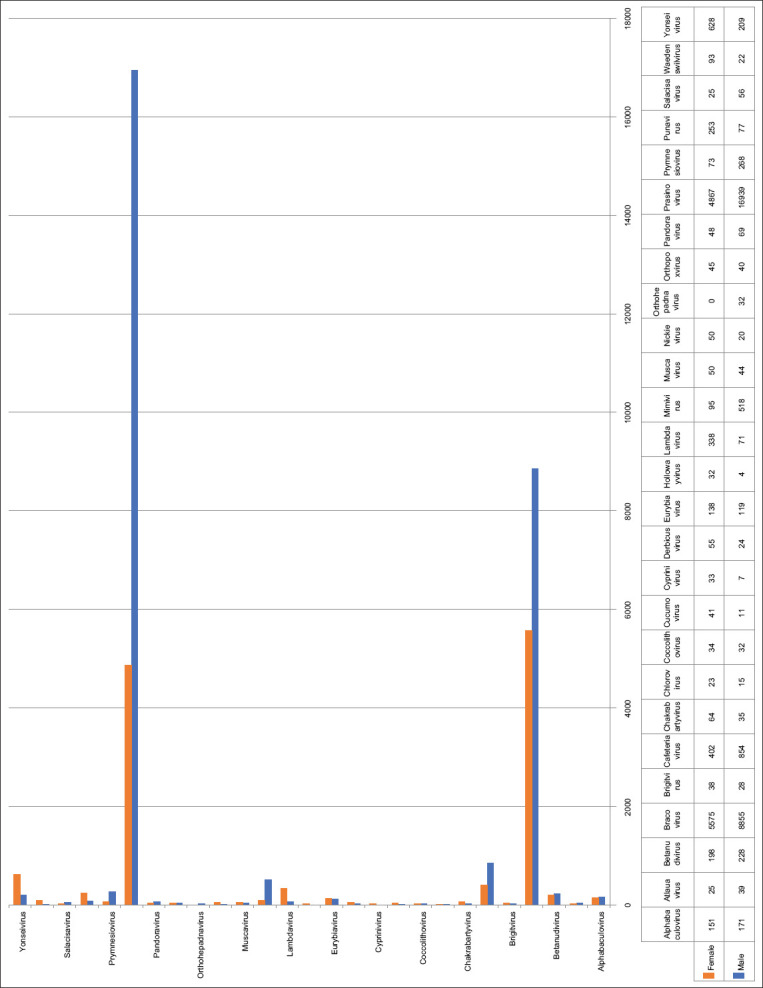
The number of viral reads at the genus level obtained from classified viruses.

The richness of each genus differed considerably in the male and female tick pools containing multiple viral sequences. *Prasinovirus* (9.4%) was found with high relative abundance in the male tick pool, followed by *Bracovirus* (4.9%). However, in the female tick pool, *Bracovirus* (2.8%) had high relative abundance followed by *Prasinovirus* (2.5%) (Figure-3).

**Figure-4:**
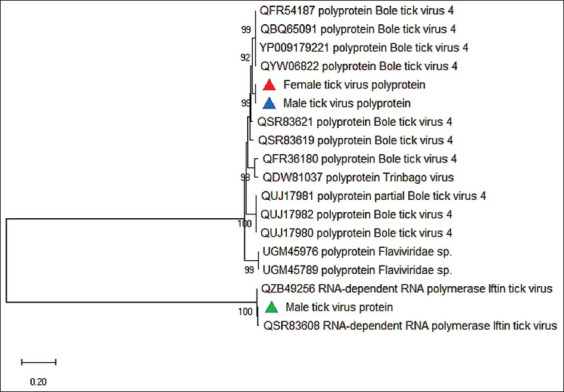
Phylogenetic tree based on viruses’ polyprotein detected in female (red triangle), male (blue triangle) ticks and protein (green triangle) in male ticks. For each sequence, we provided the accession number and virus name/strain. The tree was constructed using the Neighbor-Joining method, bootstrap test (1000 replicates), and the JTT matrix-based method [35].

*De novo* assembly of male and female samples resulted in three virus whole genomes. Among the three virus genomes in this study, two genomes have 90% similarity with unclassified Bole tick virus 4 (KR902736.1), and the third genome has 98.45% similarity with Iftin tick virus (MZ567078.1) that is *Phlebovirus* belonging to the family *Phenuiviridae* (Table-2). Therefore, our data contain two viruses. The viral genome sequences were deposited in the Zenodo data repository for both female and male tick viruses. The polyprotein tree (Figure-4) [35] was constructed with two virus species of the present study and the polyprotein from the GenBank showed the highest similarity. In the present study, polyproteins of the viruses showed the highest similarity with a Bole tick virus 4 strain detected in China (QYW06822) (Figure-4).

**Table-2:** Identified viral species with similarity to the known virus sequences from BLASTn and BLASTp databases.

Ticks (*Hyalomma dromedarii*)	Taxonomy	Species (Closest Match)	Query Cover (%)	Amino Acid/polyprotein P-Identity (%)	Contig (*n*)	Genome length
Female	Ribovirea/Unclassified	Bole tick virus 4	99	90.35/93.58	1	16305
Male	Ribovirea/Unclassified	Bole tick virus 4	99	90.36/93.76	1	15454
Male	Phenuiviridae/Phlebovirus	Iftin tick virus	99/100	98.45/99.75	1	4905

## Discussion

Whole genome sequencing (WGS) enabled us to characterize the RNA virome diversity of male and female *H. dromedarii* ticks collected from camels in Al Ain region in the UAE. Even though the WGS is a good tool for compiling and assembling genomes of different organisms, there are cases where the taxonomic assignment of sequences to the species level is not straightforward. We provided the genomes of three viruses, two of which were close to the Bole tick virus and the third showed similarity with Iftin tick virus. In the UAE, camels are important animals and have been associated with tick-borne viruses such as CCHFV [14]. Whether camels are the main reservoir of various viral disease-causing agents of humans and veterinary importance is yet to be studied. *Hyalomma dromedarii* has been found with a high prevalence in camels in various studies [25, 36]; therefore, the knowledge about the diversity of viruses in these ticks is crucial for surveillance strategies and for the management of any future tick-borne viral disease outbreak in the country. It should be mentioned that despite the fact that *H. dromedarii* is the most prevalent tick species on camels in the UAE, its microbiome is still poorly explored, despite the publication of few studies [37, 38] that characterized the bacterial communities associated with it. Therefore, the current work sheds light on unexplored viral communities, which are the other important component in its microbiome.

In this study, for molecular identification of tick species, we used the 16S rRNA sequences of *H. dromedarii* from UAE that showed 99.75% similarity with *H. dromedarii* identified from Pakistan (OL307008), 99.50% similarity with sequences of *H. dromedarii* from India (LC661680), UAE (MZ976772) [25]. The *H. dromedarii* virome revealed numerous RNA viral families. However, the data were dominated by unclassified sequences as compared to classified ones. We found a large proportion of unclassified ssRNA viruses in females compared to male ticks. This could be due to differences in the biology and feeding patterns of sexes. The amount of blood meal consumed by males is considerably less than that consumed by females and the body mass of males increases only about 1.5–2 times [39]. These findings are partially consistent with the camel tick virome characterized in a study from Saudi Arabia [40], where *Phenuiviridae* and *Nairoviridae* were detected as common families. However, in our study *Poxviridae*, *Phycodnaviridae*, and *Phenuiviridae* were the most abundant families. Furthermore, Zakham *et al*. [40] reported *Phlebovirus* as the most common genus whereas in our study the genus *Prasinovirus* was found with high relative abundance. No coronavirus was detected in either study. The differences in tick viromes between our study and Zakham *et al*. [40] could simply be due to a temporal mismatch since their samples were collected later in the year (between July and October) while ours was collected during June. Evidence from camel tick microbiomes indicates that microbial communities in ticks differ substantially during the course of a year, with notable increases in certain microbes at specific times in the year [38]. Since ecological conditions influence viruses as well, it is expected that certain groups or genera of viruses will dominate at certain times of the year. Tick-borne encephalitis virus (TBEV) in *Ixodes ricinus* ticks in Europe indicates that different tick stages, such as larvae, nymphs or adults, host a greater abundance of TBEV at different times in the year [41]. These periodic outbreaks in TBEV are associated with small mammals, such as rodents, that serve as local reservoirs of the virus during periods when no transmission occurs in humans. Some of the differences between our study and Zakham *et al*. [40] could also be because of inherent differences in regional climate and microclimate differences between the farms in UAE and Mekkah Province, Saudi Arabia [40]. It is worth noting that the camel tick virome analysis of this study showed diverse sequences related to animals and viruses, fungi, and bacteria that may be due to host-parasite relationship and their interaction with biotic and abiotic factors. The temporal and spatial ecology of tick-borne viruses are likely to be linked with the abundance of different microbial groups, as viruses are often associated with specific bacterial communities [41]. Since temporal and spatial aspects of *H. dromedarii* have not been studied, there is an urgent need to quantify patterns of high or low abundance of viruses and their diversity during the course of a year and across different sites [38]. The *Poxviridae* family was detected in our study with high relative abundance, whereas, the *Orthopoxvirus* genus was identified with some reads in both male and female ticks. *Orthopoxvirus* genus comprises a number of species that can infect animals and humans, including variola virus (the causative agent of smallpox), and monkeypox virus [42]. Recently, Orf, ecthyma contagiosum, caused by a parapox virus, has been reported in a 42-year-old man from Saudi Arabia who came into contact with an infected camel and developed a typical orf lesion [43]. Our results differed from a previous study on the characterization of tick viromes collected from dogs in China, where *Circoviridae* was detected with high abundance [44]. However, overall this study reported the highest percentage of ssRNA viruses, and similarly, we found a high abundance of unclassified ssRNA viruses in female ticks.

Whole genome sequencing provided the genomes of three viruses in the present study; two viruses showed 90.18% similarity with Bole tick virus 4 (KR902736.1, NCBI GenBank) detected in *Hyalomma asiaticum* [45] and 81.50% (MW561135.1), and 81.47% (MW561133.1) similarity with Bole tick virus 4 detected in the virome analysis of *Rhipicephalus*, *Dermacentor*, and *Haemaphysalis* ticks collected from the vegetation or engorged small ruminants from Eastern Romania [46]. For both viral genomes in our study, we found that the phylogenetic analyses placed them in clades with Bole tick virus isolates, confirming similarity and association with ticks. This relatively low level (90.18%) of sequence similarity with other GenBank genomes is likely due to the lack of viral genomes collected from ticks in the Arabian Peninsula. This demonstrates the urgent need for further studies on tick-borne viral communities in this part of the world.

The second virus showed 98.45% similarity with the Iftin tick virus (MZ567078.1, NCBI GenBank) detected in viral RNA metagenomics of *Hyalomma* ticks collected from dromedary camels in Makkah Province, Saudi Arabia [40], and 77.08% similarity with the Iftin tick virus (KM817664.1, NCBI GenBank) detected in genomic diversity of RNA viruses in arthropods reveals the ancestry of negative-sense RNA viruses [47]. A *Bracovirus* (*Polydnaviridae*) was detected as another abundant genus after *Prasinovirus*. We also identified a few sequences reads of *Reoviridae* that have been previously reported from different tick species in Asia, Europe, and Africa [21], and recently from *Ixodes uriae* [48]. Our study was limited in geographic scope to Al Ain and we suggest that future studies should consider a larger geographical distribution, which must parallel the expanding distribution of camels and camel ticks in the UAE. This would increase the chance of identifying novel tick-borne viruses that might have a potential zoonotic risk for humans or domestic animals.

In the desert ecosystems of the UAE, the camel tick life cycle can span over 2–3 years [49], depending on host availability and temperature. The expansion of camel farming due to the abundance of resources in the oil-rich Middle Eastern countries means that novel opportunities are available for the associated tick vectors [12]. Thus, the traditional camel farming industry has facilitated the geographic spread of ticks, which in turn could have promoted the maintenance and spread of tick-borne viruses in these extreme environments [12]. We found that *H. dromedarii* harbors a diversity of viruses belonging to several families of RNA and DNA viruses that can be transferred to and from neighboring countries. This diversity of tick-borne viruses needs to be properly studied to understand better the risks associated with certain groups of viruses.

## Conclusion

Characterizing a tick virome is a key factor for understanding its microbiome and knowing its pathogenic potential. In this study, we showed that the *H. dromedarii* virome contained several families and genera with some potentially pathogenic virus groups. This emphasizes the need for continued camel tick surveillance in the UAE to circumvent any future outbreak of tick-borne viruses. The current study is a pioneering work in the UAE that characterized RNA virome diversity of *H. dromedarii* and provided preliminary data that could be used as a reference for future work to detect any change in viral community composition. This work also serves as a basis for monitoring and surveillance of the prominent tick-borne virus families that are of human and animal importance.

## Data Availability

The supplementary data can be available from the corresponding author upon a reasonable request.

## Authors’ Contributions

MAA, NP, and SBM: Conceptualization. MAA, NP, BK, NS, and RSA: Methodology. NP, BK, NS, and RSA: Investigation. NS: Data curation. NS, BK, and NP: Formal analysis. NS, NP, and MAA: Visualization. MAA and SBM: Supervision and project administration. NP, NS, BK, and MAA: Writing – original draft preparation. NP, MAA, NS, SBM, and BK: Writing – review and editing. All authors have read and approved the final manuscript.
